# Prediction of Expected Years of Life Using Whole-Genome Markers

**DOI:** 10.1371/journal.pone.0040964

**Published:** 2012-07-25

**Authors:** Gustavo de los Campos, Yann C. Klimentidis, Ana I. Vazquez, David B. Allison

**Affiliations:** Section on Statistical Genetics, Department of Biostatistics, University of Alabama at Birmingham, Birmingham, Alabama, United States of America; University of Bristol, United Kingdom

## Abstract

Genetic factors are believed to account for 25% of the interindividual differences in Years of Life (YL) among humans. However, the genetic loci that have thus far been found to be associated with YL explain a very small proportion of the expected genetic variation in this trait, perhaps reflecting the complexity of the trait and the limitations of traditional association studies when applied to traits affected by a large number of small-effect genes. Using data from the Framingham Heart Study and statistical methods borrowed largely from the field of animal genetics (whole-genome prediction, WGP), we developed a WGP model for the study of YL and evaluated the extent to which thousands of genetic variants across the genome examined simultaneously can be used to predict interindividual differences in YL. We find that a sizable proportion of differences in YL—which were unexplained by age at entry, sex, smoking and BMI—can be accounted for and predicted using WGP methods. The contribution of genomic information to prediction accuracy was even higher than that of smoking and body mass index (BMI) combined; two predictors that are considered among the most important life-shortening factors. We evaluated the impacts of familial relationships and population structure (as described by the first two marker-derived principal components) and concluded that in our dataset population structure explained partially, but not fully the gains in prediction accuracy obtained with WGP. Further inspection of prediction accuracies by age at death indicated that most of the gains in predictive ability achieved with WGP were due to the increased accuracy of prediction of early mortality, perhaps reflecting the ability of WGP to capture differences in genetic risk to deadly diseases such as cancer, which are most often responsible for early mortality in our sample.

## Introduction

Agricultural and biomedical research has shown through controlled experiments and familial studies that many complex traits are highly heritable, suggesting that in principle, such traits could be predicted early in life from knowledge of individuals' genotypes. Human longevity is not an exception: empirical evidence from twin and familial studies indicate that approximately 25% of inter-individual differences in human lifespan can be attributed to genetic factors [1]–[3].

Research with model organisms offers several examples of genetic polymorphisms having a sizable effect on lifespan [4]. However, although genome-wide association studies (GWAS) and linkage scans in humans have uncovered several regions significantly associated with longevity and aging traits [5]–[9], only a few of these associations have been consistently confirmed, and our ability to predict inter-individual differences in expected Years of Life (YL) remains limited [7].

Several diseases (e.g., cancer, cardiovascular disease) and biological events (e.g., stroke, heart failure) can lead to death, and the genetic architecture (i.e., the set of genes having an effect on the trait and the ways they interact) of each of these mortality-related traits is expected to be disorder-specific. Therefore, the genetic architecture of YL is likely to include a large number, perhaps thousands, of possibly interacting genes.

Recent articles [10], [11] have suggested that the limited advances in our ability to predict complex human traits and diseases using genomic information may partially reflect the limitations of traditional GWAS to detect significant associations with complex genetic architectures. These authors have suggested that Whole Genome Prediction (WGP) may be better suited than traditional GWAS to the prediction of complex traits.

**Whole genome prediction** exploits multi-locus linkage-disequilibrium (LD) between quantitative trait loci (QTL) and genome-wide markers (e.g., SNPs) to predict inter-individual differences in a quantitative trait that are attributable to genetic factors. Unlike traditional association studies, in which the association between markers and phenotypes is tested one marker at a time, WGP uses all available markers to regress phenotype onto genomic information. This methodology was first proposed in the field of animal breeding by Meuwissen Hayes and Goddard in 2001 [12]. Since then, several simulation [12], [13] and empirical studies have demonstrated its predictive power with plant [14], [15] and animal [16]–[19] data.

More recently, research with human height showed that much of the so-called missing heritability of complex traits could be recovered using genome-wide panels of common variants [11] and, more importantly, that regression using WGP methods can improve the prediction of yet-to-be observed human phenotypes [20]. A next logical question is whether these findings apply to traits of greater medical or practical importance. Here, we: (a) extend WGP methods, which were originally developed for continuous un-censored outcomes, to accommodate censoring, a feature commonly encountered in applications with human data, (b) developed a WGP model for YL and (c) quantified the ability of this model to account for and to predict inter-individual differences in human YL that are not accounted for by major factors such as sex, Body Mass Index (BMI, kg/m^2^) and smoking.

## Materials and Methods

### Model

Many outcomes in human-health studies are either binary (e.g., presence/absence of diseases) or are subject to censoring (i.e., bounds of the outcome are known, but the exact value of outcome remains unknown). And it is well established that ignoring censoring yields biased estimates [21]. The linear models commonly used for WGP can be easily extended to accommodate binary or censored outcomes. Here, we present an extension that accommodates censoring. Similar ideas can be used to model binary outcomes as well [22].

In our WGP models, we describe YL (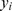, *i = 1,…,n*) as the sum of individual-specific means (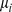) which, as we explain below, will be a function of genetic and non-genetic factors, and of a model residual (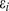) which is assumed to be a normal random variable with mean zero and variance 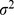; therefore 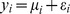. For individuals with known YL, we observe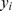; for individuals with censoring at age equal to 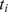, the observed event is 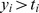. In our WGP model, expected YL (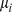) was described using a linear regression,
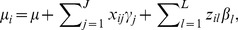
(1)which had three components: 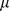, an effect common to all subjects; 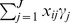, a regression component accounting for the effects of non genetic covariates (sex, smoking and BMI covariates in our application); and 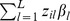, a regression on SNP genotypes 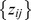 where 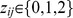 counts the number of copies of the least frequent allele at the *j^th^* SNP. By combining (1) with the normal assumptions described above, we derived the likelihood function for censored and un-censored individuals (see Methods S1 for further details).

The Bayesian model is completed by assigning a prior density to the collection of model unknowns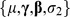. Here, we structure the prior density using a modified version of the Bayesian LASSO (BL) [23]. This model has been effectively used for WGP in plants [14], [15], [24], animals [14], [18], [19], [25] and humans [20]. We extend this model to accommodate censoring as well as effects other than those of markers. In our model, we assigned independent vague prior densities to the intercept (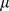) and to the effects of sex, smoking and BMI (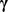). This treatment yields estimates of the effects of these non-genetic factors that are similar to those obtained with likelihood-based methods. For the remaining unknowns we adopt the prior-specification of the BL of Park and Casella [23] (see Methods S1 for further details). The joint prior-density (see expression 2 in the Methods S1) is indexed by a set of four hyper-parameters, including the prior degree of freedom and scale assigned to the residual variance (denoted as *df* and *S*, respectively), and the rate and shape parameters (dentoed as 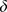 and *s,* respectively) assigned to the regularization parameter of the BL. A discussion of how these can be chosen is given in Perez et al. [26]. Here, following those guidelines, we set 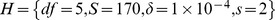. Given the characteristics of our data (sample size, number of markers and allele frequencies and observed variability on YL), these values provide priors with small influences on predictions.

### Implementation

Models were fitted using a modified version of the BLR package [27] of R [28] which handles censoring (right, left and interval) according to the model described above. In addition to BLR, R-packages bayesm [29], splines [28] and SuppDists [30] were used to implement the sampler.

### Data

(N = 5,117) were from the original (N = 1,493) and offspring (N = 3,624) cohorts of the Framingham Heart Study. Data and material distributions from this study are made in accordance with the individual consent history of each participant (see http://www.framinghamheartstudy.org/research/consentfms.html for further details about consent forms). And the current study has been approved by the Internal Review Board of University of Alabama at Birmingham (IRB Protocol Number: X090720002). The criteria for inclusion in the study included being 18 years or older at time of recruitment, having survival information as of 2007, and having complete information for covariates (sex, smoking and BMI).

Average age at entry was 37 with a standard deviation (SD) of 9.0 years. Of the participants, two thirds (N = 3,390) were censored (i.e., at the time at which survival records were defined, these individuals were still alive), 55% were female, and 36% never smoked. Mean BMI at first exam was 25.0 with a SD of 4.1 kg/m^2^. Subjects were genotyped using the Affymetrix GeneChip Human Mapping 500K Array Set. For details on the genotyping method, please refer to Framingham SHARe at the NCBI dbGaP website (http://www.ncbi.nlm.nih.gov/projects/gap/cgi-bin/study.cgi?study_id=phs000007.v3.p2). Other editing and genotyping quality control and imputation procedures were as described in Makowsky et al. [20].

### Primary Data Analysis

Using the specification of equation (3) we generated a sequence of nested models by changing the predictors included in the right-hand side of the linear predictor (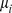). Our baseline model (denoted as M_A-0_) includes an intercept, sex, and age at entry; the latter modeled nonparametrically using a 4-df natural spline [31] with interior and boundary knots chosen using the default specifications of the natural spline (ns) function of the spline package of R [28]; with 4-df, interior knots were placed at the 25^th^, 50^th^ and 75^th^ sample percentiles of the predictor variables. We extended this model by adding smoking and BMI (also modeled nonparameterically using a 4-df natural spline [31]) this model is denoted as **M**_B-0_. Subsequently, models M_A-0_ and M_B-0_ were then extended by adding subsets of evenly spaced SNPs, from 2.5K (K = thousand) to 80K; these models were denoted as M_(.)-2.5K_, M_(.)-5K_, M_(.)-10K_, M_(.)-20K_, M_(.)-40K_ and M_(.)-80K_, where (.) was either A or B.

Models were first fitted to the entire dataset to obtain parameter estimates (estimated posterior means of effects and of variance parameters) and to evaluate the goodness of fit and the Deviance Information Criterion [32]. Subsequently, the prediction accuracy of each of the models was assessed using a 10-fold cross-validation (CV). Prediction accuracy was evaluated using two different metrics: a CV R-squared (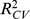) and the area under Longitudinal Receiving Operating Characteristic Curves (AUC(τ) [33]). The 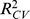 measures the proportion of inter-individual differences in years of life that can be accounted by CV-predictions, this was calculated as 
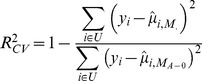
 where: 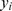 denotes observed YL of the *i^th^* individual, 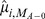 is a 10-fold CV-prediction of YL derived from model 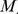 and 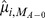 is the estimated average YL, derived from a model that only included an intercept and the effect of age at entry, which is taken here as our baseline model. This statistic can be evaluated only with subjects that have already died; therefore, in the 10-fold CV, the summation in the formula for 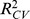 uses only data from subjects with an observed age at death. Un-censored subjects do not constitute a random sample of individuals and this may induce bias in our estimate of R-squared. Because of this, we consider a second measure of prediction performance based on longitudinal AUCs [33]. To this end we defined a sequence of thresholds (τ = 60, 65, 70, 75, 80, 85, 90, 95 YL) and for each of these thresholds we generated survival indicator variables 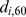,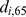,…,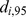 where: 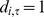 if individual *i* had YL<τ; 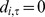 if individual *i* was still alive at time τ, and un-determined if individual *i* had an age at censoring smaller than τ. The number of individuals for which 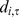 was determined (i.e., those that had known YL or age at censoring greater than τ) were 4495, 3836, 3262, 2773, 2366, 2102, 1889 and 1762 for 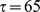,…, 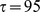, respectively. Using these survival indicator variables and CV predictions of YL (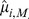) derived from the models above described we computed the AUC(τ) for every threshold using the R-package pROC [34].

### Evaluation of the effects of population structure and familial relationships on prediction accuracy

The distribution of genotypes, their allele frequency, levels of LD, etc., can be affected by factors such as population structure, admixture or familial relationships. Therefore, a certain proportion of the prediction accuracy of WGP could be attributed to those factors. To further explore this, a series of additional analysis were carried out. **First**, in order to account for population structure, we extended the model including age, sex, smoking and BMI as predictors (M_B-0_) by adding the effects of the first two principal components (PCs) derived from the same set of 80K SNPs used in M_(.)-80K_. **Second**, to quantify the relative importance of familial relationships on prediction accuracy we carried out two additional analyses: (a) we extended M_B-0_ by adding an effect representing a regression on the pedigree. This was done using the standards of the additive infinitesimal model of quantitative genetics [35], and this model is denoted as M_B-PED_. And (b) we fitted models M_A-0K_, M_B-0K_ and M_B-80K_ in a 10fold CV where entire families, as opposed to individuals, were assigned to folds; therefore, in this CV predictions are derived from nominally-unrelated individuals.

## Results

### Full data analysis

Using M_B-0_, we estimated an average (± posterior SD) difference in YL between females and males of 3.1 (±0.42) years and between smokers and nonsmokers of −4.1 (±0.44) years. Using estimates from M_B-0_, we computed the expected YL of a nonsmoking 35-year-old by sex and BMI; the results are displayed in (Figure S1). Expected YL was greatest within the range 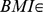 [20], [25]; extreme BMI values, lower than 20 or higher than 25, were associated with a decrease in YL. Using M_B-0_ we estimate an expected decrease in YL of 0.43 year per extra unit of BMI in the range 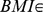 [25], [40]. Overall, these patterns are in agreement with what has been reported previously for the effect of sex [36]–[38], smoking [36], [39] and BMI [21], [36] on YL.

Table 1 shows estimates of residual variance and DIC by model. The intercept-only model (not included in Table 1) yielded an estimate of variance of YL of 135, and the estimated residual variance of M_A-0_ was 104.1; therefore, approximately 23%, computed as 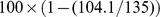, of observed variability in YL in our dataset can be explained by differences in age at entry and sex. Model M_B-0_ yielded an estimate of residual variance of 98.7, indicating that BMI and smoking accounted for about 5% of inter-individual differences in YL that were not accounted for by age at entry and sex; this was computed as 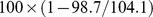. Adding SNPs to M_A-0_ or M_B-0_ resulted in a marked increase in goodness of fit, and this is reflected in a substantial reduction in the estimated residual variance (Table 1). For instance, M_B-80K_ yielded an estimate of the residual variance that was 65% smaller than that of the M_B-0K_, computed as 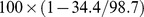.

**Table 1:** Estimated posterior mean of residual variance and Deviance Information Criterion (DIC, ‘smaller is better’) by number of SNPs (rows) and nongenetic covariates (columns) included in the model.

	Residual	Variance*	Deviance Information Criterion (DIC)	
Thousands of SNPs in the Model	Age+Sex	Age+Sex+BMI+Smoking	Age+Sex	Age+Sex+BMI+Smoking
0	104.1	98.7	14,744	14,625
2.5	79.7	75.5	14,268	14,158
5.0	68.3	64.1	14,130	14,007
10.0	57.1	554.5	13,951	13,845
20.0	48.1	46.2	13,772	13,673
40.0	40.3	39.6	13,540	13,479
80.0	34.6	34.4	13,337	13,289

* : Posterior mean of the residual variance, the estimate of this parameter can be regarded as a proxy of goodness of fit to the data used to train the model.

Due to the curse of dimensionality [40], the increase in goodness of fit achieved by adding SNPs to the model may reflect genetic variability captured by SNPs, over-fitting, or a combination of both. However, DIC, a model comparison criterion that balances goodness of fit and model complexity, decreased monotonically with the number of SNPs, suggesting that information is being added as marker density increases.

### Evaluation of prediction accuracy in cross validation

**Figure 1:**
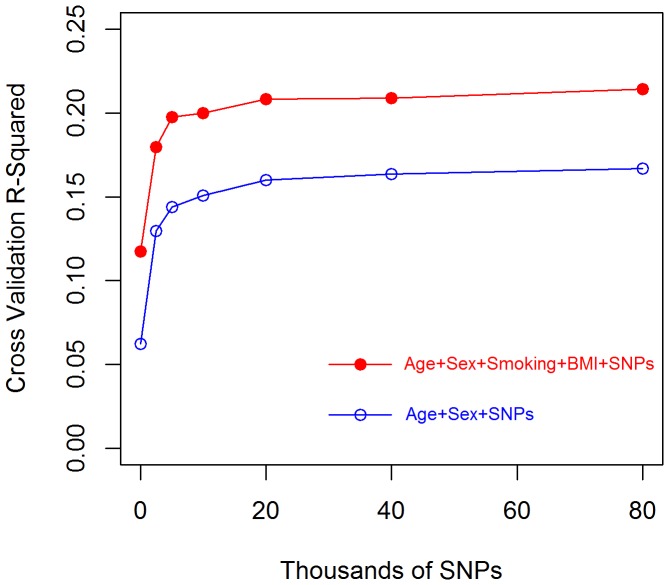
Cross-validation R-squared (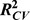) by number of markers and model. Circles represent the 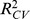 obtained in a 10-fold CV.

Figure 1 shows estimated 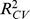 versus marker density (from 0 to 80K) by model. The 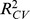 of a model including age at entry and sex, 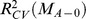, was approximately 6%. The addition of smoking and BMI resulted in a doubling of 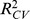, from 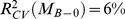 to 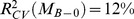; as expected, the addition of smoking and BMI increased prediction accuracy by a sizable amount. Prediction accuracy increased monotonically with the number of markers both in models with and without BMI and smoking covariates. These results confirm that markers are capturing information about expected YL that cannot be predicted using major factors such as age at entry, sex, smoking and BMI. Using 80K markers, we were able to increase 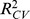 from 6% to 11% for the model without smoking and BMI (M_A-(.)_) and from 12% to 21% for the model including smoking and BMI (M_B-(.)_). (Table S1) shows 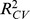 for models M_A-0_, M_B-0_ and M_B-80K_ by fold of the CV. The variability in 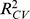 across folds reflects uncertainty about our estimates due to sampling of training and testing datasets. Although we observed an overall superiority of M_B-0_ over M_A-0_ this superiority did not occur in every fold of the CV. However, M_B-80K_ outperformed models without SNP information (M_A-0_ and M_B-0_) consistently across folds indicating that SNPs are capturing important and consistent patterns of variability in human lifespan.

**Figure 2:**
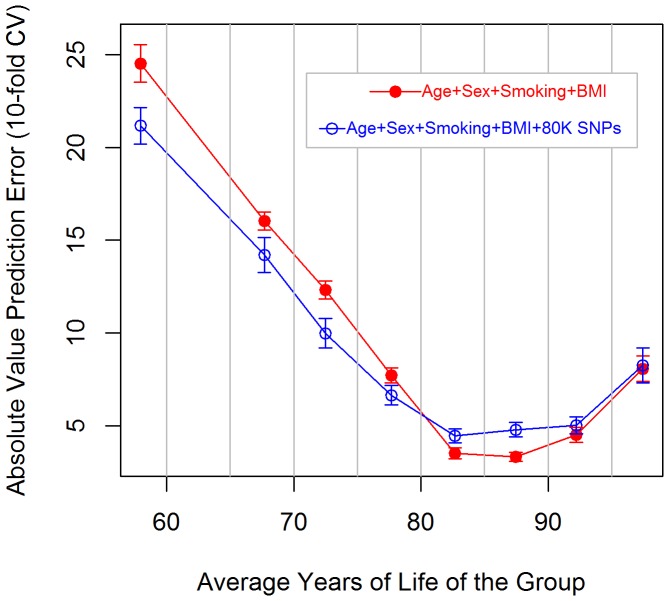
Absolute value CV prediction error versus range of YL. Circles represent the average absolute value prediction error for each group of YL (YL≤65, 65<YL≤70,..,YL>95); and vertical bars represent the 95% confidence interval defined by the average absolute value prediction error ±1.96×SE.

The above results indicate that markers can explain a sizable proportion of inter-individual differences in YL that are not accounted for by age at entry, sex, smoking and BMI. To obtain further insights into the source of this improvement in prediction accuracy, we present in Figure 2 the average absolute value of the CV prediction error (from the 10-fold CV) and its SE by range of YL for models M_B-0K_ and M_B-80K_. As expected, for both models, the absolute value prediction error was lowest for people dying around median age (80 YL) and increased for people dying early or late in life. Predictions derived from model M_B-80K_ were much more accurate than those of M_B-0_ for the prediction of YL of people dying early in life; however, the prediction accuracy of the model with markers was slightly higher than that of M_B-80K_ for subjects dying at intermediate ages. This suggests that the overall higher predictive ability of M_B-80K_ is due mostly to improvements in prediction of early mortality.

**Figure 3:**
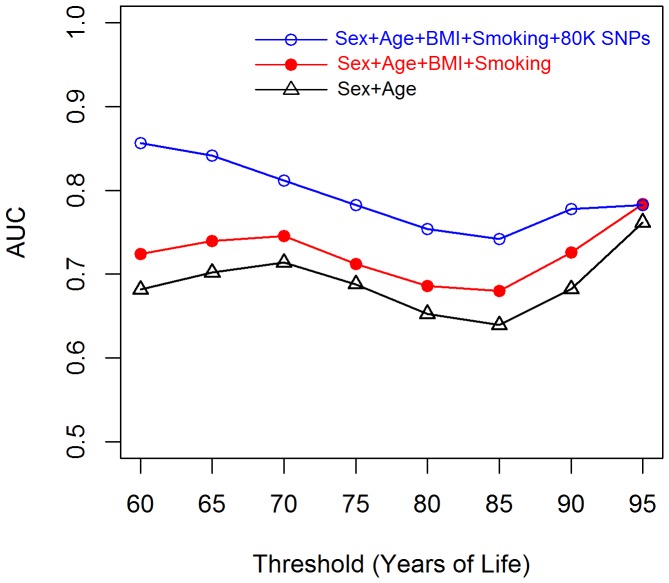
Area under the receiving operating characteristic curve (AUC) for survival status define at different time points (60, 65,…,95 years of life) and three models that differed on the predictor variables used to predict expected years of life.

Figure 3 shows the AUC (vertical axis) for models M_A-0_, M_B-0_, and M_B-80K_ for each of the 8 thresholds (horizontal axis). Adding BMI and smoking information to a model that included sex and age (M_B-0_ vs M_A-0_) resulted in an increase in AUC(τ) of roughly 5–7%. When 80 thousand SNPs (M_B-80K_) were added to a model that included age, sex, smoking and BMI as covariates we observed a substantial increase in classification performance for prediction of early stage survival status (relative to M_B-0_, M_B-80K_ yielded an increase in AUC(60) of 18%), a more modest increase in AUC(τ) for survival status at ages 65–90 (M_B-80K_ outperformed M_B-0_ by about 14% for AUC(65) and by 7–10% for AUC(70)–AUC(90)), and no change in AUC(95). These results are consistent with those observed with 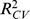 in that they indicate that genomic information can increase the prediction accuracy of lifespan, mostly due to an increase in the prediction of early mortality. Table S1 shows estimates of AUC(τ) for models M_A-0_, M_B-0_ and M_B-80K_ by fold of the CV. Similar to what we observed for 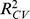, although we found an overall superiority in the classification performance of M_B-0_ relative to that of M_A-0_ such superiority was not consistently observed in every fold. However, for early and intermediate survival status (τ≤85) model M_B-80K_ had a classification performance that was consistently higher than that of models without genetic information (M_A-0_ and M_B-0_). For late mortality (τ>85) such superiority was not consistently observed across folds.

### Effects of population structure

The estimated 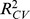 of model M_B-GWPC_ was 15.77%, this is roughly half the way from the 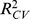 of model M_B-0_ (11.45%) and that of model M_B-80K_ (21.40%). Results for AUC showed similar patterns. This indicates that a sizable proportion of inter-individual differences in YL could be attributed to genetic differences associated to population structure. On the other hand, the fact that the 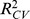 of model M_B-80K_ was 37% higher than that of model M_B-GWPC_ suggests that genetic factors beyond those associated with population structure account for a sizable proportion of inter-individual differences in YL.

### Effects of familial relationships

Model M_B-PED_, which including age at entry, sex, BMI, smoking and pedigree information showed clear signs of over-fitting (the posterior mean of the residual variance was 11.1, compared to 34.4 for model M_B-80K_) and, consequently, had a very poor predictive performance; even worse than our baseline model (M_A-0_). This is most likely to occur because of two reasons. First, the pedigree is very sparse, with 37% of nominally un-related individuals and most of the remaining individuals coming from relatively small nuclear families (74% of individuals were in families with 3 or less members). Additionally, in the great majority of nuclear families the offspring have censored YL. Therefore, in this dataset the amount of familial information available for prediction is very limited. To further illustrate this, we counted for every subject in the 10-fold CV where individuals were randomly assigned to folds the number of close-relatives (father, mother, offspring or full-sib) which were used for prediction (i.e., those which were assigned to a different fold). We found that in our CV 41.6 of the observations were predicted without having any direct relative in the training dataset (i.e. in other folds) and 70.75 were predicted without having any un-censored direct relative available for training. Only 10% of individuals had 3 or more direct relatives in the training datasets, and no-one had 3 or more direct relative with observed YL assigned to a different fold.

Our second approach to quantify the relative importance of family relationships on prediction accuracy consisted on fitting models M_A-0_, M_B-0_ and M_B-80K_ in a10-fold CV where entire families, as opposed to individuals, were assigned to folds. Such setting guarantees that no-direct relatives are used for prediction. The 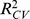 obtained in this new CV were very similar (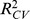 were 11.9% and 22.3% for M_B-0_ and M_B-80K_, respectively) to the ones we obtained when subjects, as opposed to entire families, were assigned to folds (here, 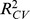 were 11.9% and 22.3% for M_B-0_ and M_B-0_, respectively). Combining all these results we conclude that in our analysis familiar relationships were not a major factor explaining the prediction accuracy obtained with WGP.

### Prediction accuracy and causes of mortality

**Figure 4:**
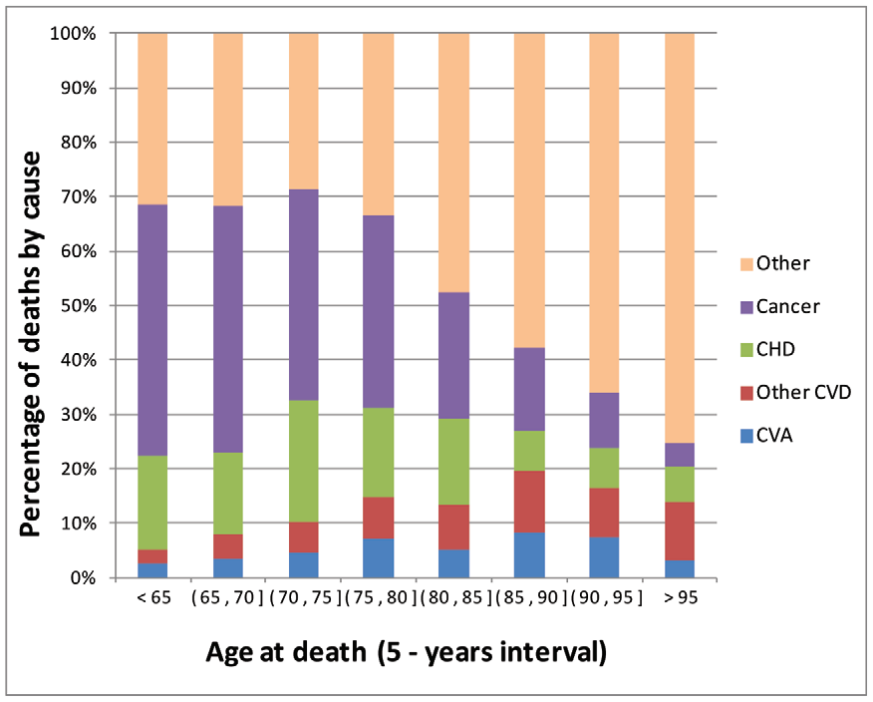
Proportion of deaths by cause, and range of age at death. Causes included cancer, coronary heart disease (CHD), cardio-vascular accident (CVA), other cardio-vascular diseases (Other CVD) and other causes.

Our results suggest that genomic information can enhance prediction of lifespan, mostly by improving prediction of early mortality. This can be due to several factors, one of which may be that SNPs are capturing genetic risk to certain diseases that are most responsible for early mortality. Figure 4 presents the distribution of death by cause and range of age at death in the Framingham sample. Cancer was the leading cause of death for people dying early in life, and the relative importance of cancer as a cause of death declined with increasing YL. On the other hand, the relative importance of other causes of death was much higher for people dying at older ages.

## Discussion

Familial studies suggest that roughly 25% of the inter-individual differences in YL can be attributed to genetic factors [7]. Although linkage and association studies have reported several variants associated with human lifespan and aging-related traits [6], [8], [9], [41], the individual effects of these variants is usually small and our ability to use genetic information to predict human lifespan remains very limited. Recent studies [10], [11], [20] suggest that WGP is effective at predicting complex traits. Here, we developed a WGP model for the prediction of YL and evaluated its predictive power using data from the Framingham longitudinal study.

When genetic markers were added to a model accounting for age at entry, sex, smoking, and BMI, the increase in 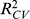 obtained by adding 80K SNPs (∼9–10% of inter-individual differences in YL) was greater than the increase obtained by adding smoking and BMI (∼6% of inter-individual differences in YL), indicating that genetic markers are making a relatively important contribution to predictive ability. Similar results were obtained when prediction accuracy was evaluated using longitudinal AUC's.

As anticipated, our results suggest that the genetic basis of YL involves a large number of variants. The observation that DIC and prediction accuracy improved with marker density suggests that a large number of markers spread across the genome are needed to account for differences at QTLs affecting YL, and this is consistent with what one would expect for a trait that conforms to an “infinitesimal” model [42], [43]. This pattern is also consistent with empirical evidence obtained for traits that conform to the infinitesimal model, such as human height [20] or production traits in dairy cattle [19].

Our results are also consistent with those of Yashin et al. [44] who, using a subset of the dataset used here (1,173 individuals of the original cohort), found that a sizable proportion of inter-individual differences in YL (20% in the training dataset) can be explained by the joint influence of 168 small-effect genetic variants which were pre-selected using *p-values* derived from single-marker regressions. Although the study by Yashin et al. [44] and the one presented here both suggest that a large number of variants is needed to account for interindividual differences in YL, the two studies differ in many respects: (a) our study uses a larger sample size (N = 5,117, versus N = 1,173) and incorporates both uncensored and censored observations, (b) unlike the Yashin study, where markers were pre-selected using statistics derived from single marker regression, here we used a much larger number of markers (up to 80K), spread along the whole genome, (c) although the two studies used an additive linear score to predict YL, the two scores are different. In the Yashin study the score consist of a sum of so-called “longevity alleles”, while in our study the predictive score is a weighted sum of allele dosage, with weights given by estimates of marker effects, (d) in some of our models we account for the effects of BMI and smoking, while these covariates were not accounted for in the Yashin study. Finally (d) in our study we focused on prediction accuracy of yet-to be observed outcomes, while the study by Yashin et al. reports the proportion of interindividual differences in YL that could be accounted for in the same dataset that was used to derive the predictive score. Nevertheless, despite the differences in the datasets and methods used, both studies provide consistent evidence that an important proportion of differences in YL can be predicted using genomic information and that capturing those patterns requires considering a large number of small-effects variants.

In addition to demonstrating that a sizable proportion differences in YL can be predicted using genomic information, we found that most of the gains in prediction accuracy obtained with use of genetic information came from increased accuracy of prediction of early mortality. Further examination of the distribution of causes of death by age at death reveled that cancer was the leading cause of death for people dying early in life. Therefore, a possible explanation of our results is that the ability of our WGP to capture cancer risk (indirectly through YL) was higher than for other death-related disorders. Further studies, using disorder-specific responses (e.g., presence/absence or onset of cancer) and case-control datasets will be needed to confirm this conjecture.

The Framingham dataset has a familial design and exhibits some level of population structure, much of which can be described through PCA of genome-wide SNPs. Whole-Genome Prediction exploits multi-locus LD between markers and QTL. These patterns of LD are likely to change across sub-groups in the population and because of this, models fitted using WGP cannot be regarded as ‘universal equations’. The validity across sub-groups of the patterns captured by a WGP model will depend on the extent to which genetic features (e.g., stratification) present in training samples are also present in those used for validation.

Including the first two marker-derived PCs increased prediction accuracy markedly, indicating that YL covariates with ancestry, as described by the first 2 PCs. However, the level of prediction accuracy attained by models using the first two marker-derived PCs was substantially lower than that of the model using 80K genome-wide SNPs, suggesting that the genetic factors affecting YL cannot be fully described by features such as population structure. The effects of familial relationships on the prediction accuracy of WGP are well established [13], [20]. However, in our study, the pedigree is relatively sparse and when families with more than one subject exist the offspring are highly likely to be censored; therefore, familiar relationships are not very informative to begin with, explaining why in this study familial relationships did not show a strong effect on the prediction accuracy of WGP.

## Supporting Information

Methods S1 **Describes the Bayesian model used.**
(DOCX)

Figure S1 **Estimated expected years of life versus Body Mass Index (BMI) by sex (estimates derived from a model which included sex, age at entry, smoking and BMI as predictors).**
(TIFF)

Table S1 **Cross-validation R-squared and Area Under Longitudinal Receiver Operating Characteristic Curves by model and fold of a 10-fold cross-validation.**
(DOC)
