# Supplementary material for: Prediction of Expected Years of Life Using Whole-Genome Markers
Source: PLoS One. 2012 Jul 25;7(7):e40964. doi: 10.1371/journal.pone.0040964 (PMC3405107; doi:10.1371/journal.pone.0040964)
Supplement: Methods S1 — Describes the Bayesian model used. (DOCX) [file pone.0040964.s001.docx]

Methods S1

**Whole Genome Regression & Prediction of Censored Traits Using and Extended Version of the Bayesian LASSO**

In our WGP model, we describe the conditional expectation function of the response ($y_{i}$, *i=1,…,n*) as the sum of individual-specific means ($\mu_{i}$) and of a model residual ($\varepsilon_{i}$) which is assumed to be a normal random variable with mean zero and variance $\sigma^{2}$; therefore $y_{i}=\mu_{i}+\varepsilon_{i}$. Here,

$\mu_{i}=\mu+\sum_{j=1}^{J} x_{ij}\gamma_{j}+\sum_{l=1}^{L} z_{il}\beta_{l}$, (1)

is a linear predictor that has three components: $\mu$, an effect common to all subjects; $\sum_{j=1}^{J} x_{ij}\gamma_{j}$, a regression component accounting for the effects of non genetic covariates (sex, smoking and BMI covariates in our application) ; and $\sum_{l=1}^{L} z_{il}\beta_{l}$, a regression on SNP genotypes $\left\{z_{ij}\right\}$ where $z_{ij}\in\left\{0,1,2\right\}$ counts the number of copies of the least frequent allele at the *j^th^* SNP. Combining the above-described assumptions we obtain the likelihood function un-censored data points

$p\left(\left.y_{i}\right|\mu_{i},\sigma_{2}\right)=\left(2\pi\sigma^{2}\right)^{-\frac{1}{2}}\exp\left\{-\frac{\left(y_{i}-\mu_{i}\right)^{2}}{2\sigma^{2}}\right\}$ . (2)

and that of surviving subjects

$p\left(\left.y_{i}\geq t_{i}\right|\mu_{i},\sigma_{2}\right)=\int_{t_{i}}^{\infty} \left(2\pi\sigma^{2}\right)^{-\frac{1}{2}}\exp\left\{-\frac{\left(y_{i}-\mu_{i}\right)^{2}}{2\sigma^{2}}\right\}\partial y_{i}=\Phi\left(\frac{\mu_{i}-t_{i}}{\sigma}\right)$, (3)

where $t_{i}$ represent age at censoring of the *i^th^* individual and $\Phi\left(\cdot\right)$ is the cumulative distribution function of a standard normal random variable. Expression (3) represents the likelihood function of a right-censored observation. Similar expression can be derived for left and interval censored observations; herein we focus on right-censored observation only.

Combining equations (1)-(3) and under conditional independence, we obtain the conditional density of the data given effects and the residual variance:

| ***Likelihood:*** | $\begin{matrix} p\left(\left.\mathbf{y}\right\vert\mu,\mathbf{\gamma,\beta},\mathbf{t},\sigma_{2}\right)=\left\{\prod_{i\in U} N\left(\left.y_{i}\right\vert\mu+\sum_{j=1}^{J} x_{ij}\gamma_{j}+\sum_{l=1}^{L} z_{il}\beta_{l},\sigma^{2}\right)\right\} \\ \text{ }\times\left\{\prod_{i\in C} \Phi\left(\frac{t_{i}-\mu-\sum_{j=1}^{J} x_{ij}\gamma_{j}-\sum_{l=1}^{L} z_{il}\beta_{l}}{\sigma}\right)\right\} \end{matrix}$ (4) |
| --- | --- |

where $\mathbf{y}=\left\{y_{i}\right\}$, $\mathbf{\gamma}=\left\{\gamma_{j}\right\}$ , $\mathbf{\beta}=\left\{\beta_{l}\right\}$ and $\mathbf{t}=\left\{t_{i}\right\}$ are vectors of data effects and age at censoring, respectively, and $i\in U$and $i\in C$ indicate that the product runs over the set of un-censored and censored records, respectively.

Bayesian inference is based on the posterior density of the unknowns $\left\{\mu,\mathbf{\gamma,\beta},\sigma_{2}\right\}$ given the data. Following Bayes’ theorem, this is proportional to the conditional density of the data, given by (4), times the prior density of model unknowns. Here, we structure the prior density using a modified version of the Bayesian LASSO [1]. We extend this model to accommodate censoring, according to (4), as well as effects other than those of markers. Specifically, we assign independent vague prior densities to the intercept ($\mu$) and to the effects of sex, smoking and BMI ($\mathbf{\gamma}$). This treatment yields estimates of the effects of these non-genetic factors that are comparable to those obtained with likelihood-based methods. Following standard assumptions of Bayesian regression models, the residual variance is assigned a scaled-inverse Chi-square density, $\chi^{-2}\left(\left.\sigma_{2}\right|df,S\right)$, with degree of freedom and scale parameter $df$ and $S$, respectively. The prior density of marker effects is structured using the specification used in the Bayesian LASSO of Park and Casella [1], here,

$p\left(\left.\mathbf{\beta},\mathbf{\tau}^{2},\lambda^{2}\right|H,\sigma^{2}\right)=p\left(\left.\mathbf{\beta}\right|\mathbf{\tau}^{2}\sigma^{2}\right)p\left(\left.\mathbf{\tau}^{2}\right|\lambda^{2}\right)p\left(\left.\lambda^{2}\right|r,s\right)=\left\{\prod_{l=1}^{L} N\left(\left.\beta_{l}\right|0,\tau_{l}^{2}\sigma_{2}\right)Exp\left(\left.\tau_{l}^{2}\right|\lambda^{2}\right)\right\}G\left(\left.\lambda^{2}\right|r,s\right)$,

were: $N\left(\left.\beta_{l}\right|0,\tau_{l}^{2}\sigma_{2}\right)$ is a normal density assigned to the effect of the *l^th^* marker, centered at zero and with marker-specific prior variance $\tau_{l}^{2}\sigma_{2}$. In the next level of the hierarchy, the prior variance of marker effects are assigned IID exponential priors, $Exp\left(\left.\tau_{l}^{2}\right|\lambda^{2}\right)$, each indexed by a regularization parameter, $\lambda^{2}$, which controls the shape of this prior and, with this, the amount of shrinkage of estimates of marker effects. In the last level of the hierarchy the regularization parameter is assigned a Gamma prior density. Such treatment allows inferring this important parameter from the data. Collecting the assumptions above described, the joint prior density becomes:

***Prior***: $p\left(\left.\mu,\mathbf{\gamma,}\sigma^{2},\mathbf{\beta},\mathbf{\tau}^{2}\lambda^{2}\right|H\right)\propto\chi^{-2}\left(\left.\sigma_{2}\right|df,S\right)\left\{\prod_{l=1}^{L} N\left(\left.\beta_{l}\right|0,\tau_{l}^{2}\sigma_{2}\right)Exp\left(\left.\tau_{l}^{2}\right|\lambda^{2}\right)\right\}G\left(\left.\lambda^{2}\right|r,s\right)$ (5)

Expression (5) is indexed by a set of four hyper-parameters$H=\left\{df,S,\delta,s\right\}$, a discussion of how these can be chosen is given in Perez et al.[2].

Following Bayes’ theorem the posterior density is proportional to the product of the conditional density of the data (4) and the prior (5); therefore,

$$\begin{matrix} p\left(\left.\mu,\mathbf{\gamma,\beta},\mathbf{\tau}^{2},\sigma_{2},\lambda^{2}\right|\mathbf{y},\mathbf{t}\right)=\left\{\prod_{i\in U} N\left(\left.y_{i}\right|\mu+\sum_{j=1}^{J} x_{ij}\gamma_{j}+\sum_{l=1}^{L} z_{il}\beta_{l},\sigma^{2}\right)\right\} \\ \text{ }\times\left\{\prod_{i\in C} \Phi\left(\frac{t_{i}-\mu-\sum_{j=1}^{J} x_{ij}\gamma_{j}-\sum_{l=1}^{L} z_{il}\beta_{l}}{\sigma}\right)\right\} \\ \text{ }\times\chi^{-2}\left(\left.\sigma_{2}\right|df,S\right)\left\{\prod_{l=1}^{L} N\left(\left.\beta_{l}\right|0,\tau_{l}^{2}\sigma_{2}\right)Exp\left(\left.\tau_{l}^{2}\right|\lambda^{2}\right)\right\}G\left(\left.\lambda^{2}\right|r,s\right)\text{ (6)} \end{matrix}$$

This posterior density does not have closed form; however, using data-augmentation we can draw samples from the posterior density using a Gibbs sampler. A modified version of the BLR package [27] that handles right, left and interval censored was developed and it is available upon request to the corresponding author.
