# Supplementary figures and images for: Prediction of Expected Years of Life Using Whole-Genome Markers
Source: PLoS One. 2012 Jul 25;7(7):e40964. doi: 10.1371/journal.pone.0040964 (PMC3405107; doi:10.1371/journal.pone.0040964)

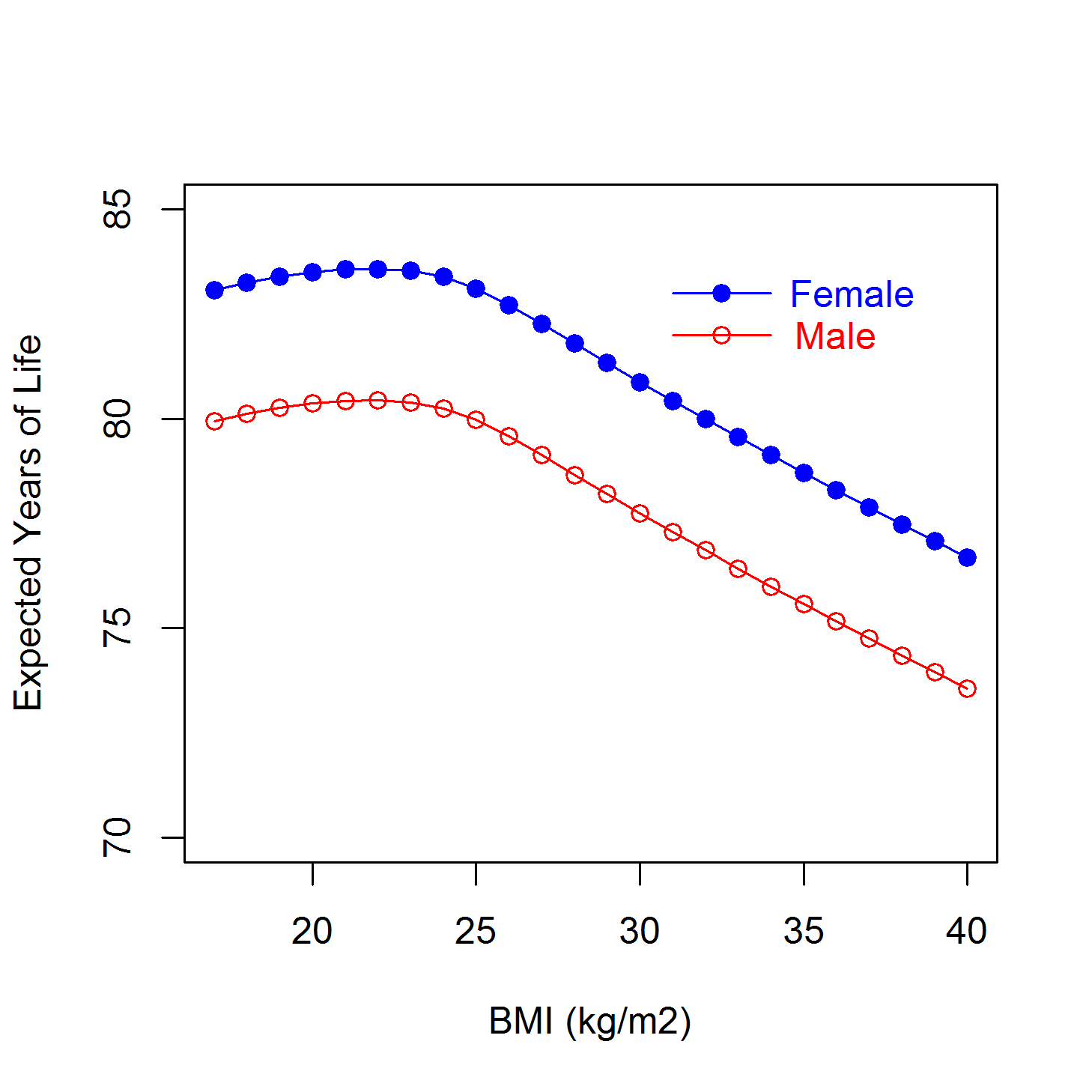

Supplement: Figure S1 — Estimated expected years of life versus Body Mass Index (BMI) by sex (estimates derived from a model which included sex, age at entry, smoking and BMI as predictors). (TIFF) [file pone.0040964.s002.tif]
