# Supplementary material for: Prediction of Expected Years of Life Using Whole-Genome Markers
Source: PLoS One. 2012 Jul 25;7(7):e40964. doi: 10.1371/journal.pone.0040964 (PMC3405107; doi:10.1371/journal.pone.0040964)
Supplement: Table S1 — Cross-validation R-squared and Area Under Longitudinal Receiver Operating Characteristic Curves by model and fold of a 10-fold cross-validation. (DOC) [file pone.0040964.s003.doc]

**Supplementary Data**

**Table S1**. Cross-validation R-squared and Area Under Longitudinal Receiver Operating Characteristic Curves (AUC(τ) for τ=60,65,…,90,95) by model and fold of a 10-fold cross-validation. Model M_A-0_ includes sex and age at entry as predictors; model M_B-0_ includes sex, age at entry, smoking and body-mass index (BMI) as predictors and model M_B-80K_ includes sex, age at entry, smoking, BMI and 80 thousand SNPs as predictors.

| Statistic | Model | Fold=1 | Fold=2 | Fold=3 | Fold=4 | Fold=5 | Fold=6 | Fold=7 | Fold=8 | Fold=9 | Fold=10 | Average |
| --- | --- | --- | --- | --- | --- | --- | --- | --- | --- | --- | --- | --- |
|  | M_A-0_ | 0.089 | 0.083 | 0.099 | 0.073 | 0.016 | 0.013 | 0.038 | 0.008 | 0.103 | 0.086 | 0.061 |
| R-Squared | M_B-0_ | 0.160 | 0.077 | 0.163 | 0.130 | 0.093 | 0.038 | 0.083 | 0.103 | 0.165 | 0.142 | 0.115 |
|  | M_B-80K_ | 0.258 | 0.216 | 0.266 | 0.224 | 0.233 | 0.176 | 0.217 | 0.192 | 0.170 | 0.194 | 0.215 |
|  | M_A-0_ | 0.805 | 0.792 | 0.851 | 0.506 | 0.659 | 0.419 | 0.652 | 0.538 | 0.635 | 0.690 | 0.655 |
| AUC(60) | M_B-0_ | 0.780 | 0.791 | 0.866 | 0.602 | 0.694 | 0.598 | 0.704 | 0.652 | 0.696 | 0.749 | 0.713 |
|  | M_B-80K_ | 0.868 | 0.885 | 0.921 | 0.922 | 0.815 | 0.731 | 0.869 | 0.886 | 0.816 | 0.849 | 0.856 |
|  | M_A-0_ | 0.752 | 0.763 | 0.827 | 0.642 | 0.526 | 0.654 | 0.687 | 0.656 | 0.774 | 0.750 | 0.703 |
| AUC(65) | M_B-0_ | 0.745 | 0.755 | 0.851 | 0.725 | 0.615 | 0.646 | 0.763 | 0.741 | 0.778 | 0.792 | 0.741 |
|  | M_B-80K_ | 0.824 | 0.889 | 0.886 | 0.898 | 0.826 | 0.727 | 0.920 | 0.851 | 0.785 | 0.846 | 0.845 |
|  | M_A-0_ | 0.792 | 0.795 | 0.870 | 0.697 | 0.595 | 0.628 | 0.761 | 0.674 | 0.721 | 0.714 | 0.725 |
| AUC(70) | M_B-0_ | 0.770 | 0.762 | 0.831 | 0.758 | 0.705 | 0.598 | 0.728 | 0.762 | 0.810 | 0.767 | 0.749 |
|  | M_B-80K_ | 0.808 | 0.843 | 0.847 | 0.843 | 0.830 | 0.716 | 0.827 | 0.872 | 0.776 | 0.801 | 0.816 |
|  | M_A-0_ | 0.759 | 0.733 | 0.711 | 0.683 | 0.687 | 0.576 | 0.695 | 0.633 | 0.735 | 0.707 | 0.692 |
| AUC(75) | M_B-0_ | 0.802 | 0.724 | 0.705 | 0.691 | 0.708 | 0.600 | 0.690 | 0.713 | 0.788 | 0.728 | 0.715 |
|  | M_B-80K_ | 0.807 | 0.791 | 0.761 | 0.797 | 0.813 | 0.743 | 0.800 | 0.771 | 0.786 | 0.771 | 0.784 |
|  | M_A-0_ | 0.682 | 0.674 | 0.681 | 0.632 | 0.669 | 0.581 | 0.627 | 0.579 | 0.707 | 0.696 | 0.653 |
| AUC(80) | M_B-0_ | 0.761 | 0.660 | 0.687 | 0.654 | 0.681 | 0.597 | 0.652 | 0.690 | 0.758 | 0.712 | 0.685 |
|  | M_B-80K_ | 0.816 | 0.722 | 0.760 | 0.686 | 0.768 | 0.738 | 0.788 | 0.750 | 0.762 | 0.749 | 0.754 |
|  | M_A-0_ | 0.642 | 0.661 | 0.660 | 0.570 | 0.692 | 0.564 | 0.689 | 0.590 | 0.716 | 0.632 | 0.641 |
| AUC(85) | M_B-0_ | 0.722 | 0.670 | 0.679 | 0.605 | 0.749 | 0.572 | 0.697 | 0.685 | 0.754 | 0.671 | 0.680 |
|  | M_B-80K_ | 0.791 | 0.744 | 0.745 | 0.672 | 0.771 | 0.709 | 0.781 | 0.744 | 0.736 | 0.738 | 0.743 |
|  | M_A-0_ | 0.640 | 0.704 | 0.748 | 0.656 | 0.736 | 0.632 | 0.729 | 0.599 | 0.728 | 0.696 | 0.687 |
| AUC(90) | M_B-0_ | 0.699 | 0.677 | 0.780 | 0.701 | 0.820 | 0.657 | 0.758 | 0.686 | 0.775 | 0.731 | 0.728 |
|  | M_B-80K_ | 0.807 | 0.799 | 0.812 | 0.744 | 0.788 | 0.752 | 0.785 | 0.771 | 0.754 | 0.773 | 0.778 |
|  | M_A-0_ | 0.699 | 0.775 | 0.899 | 0.741 | 0.733 | 0.807 | 0.834 | 0.598 | 0.786 | 0.782 | 0.765 |
| AUC(95) | M_B-0_ | 0.716 | 0.725 | 0.898 | 0.678 | 0.790 | 0.789 | 0.863 | 0.775 | 0.804 | 0.744 | 0.778 |
|  | M_B-80K_ | 0.763 | 0.824 | 0.874 | 0.783 | 0.772 | 0.825 | 0.827 | 0.723 | 0.801 | 0.724 | 0.792 |
